# Impact of interstitial elements on the stacking fault energy of an equiatomic CoCrNi medium entropy alloy: theory and experiments

**DOI:** 10.1080/14686996.2022.2080512

**Published:** 2022-08-30

**Authors:** Igor Moravcik, Martin Zelený, Antonin Dlouhy, Hynek Hadraba, Larissa Moravcikova-Gouvea, Pavel Papež, Ondřej Fikar, Ivo Dlouhy, Dierk Raabe, Zhiming Li

**Affiliations:** a Institute of Materials Science and Engineering, Faculty of Mechanical Engineering, Brno University of Technology, NETME Centre, Brno, Czech Republic; b Microstructure Physics and Alloy Design, Max-Planck-Institut für Eisenforschung GmbH, Düsseldorf, Germany; c Institute of Physics of Materials of the Czech Academy of Sciences, Brno, Czech Republic; d School of Materials Science and Engineering, Central South University, Changsha, China

**Keywords:** *ab initio* calculations, interstitials, medium entropy alloy, scanning transmission electron microscopy, stacking fault energy, strengthening

## Abstract

We investigated the effects of interstitial N and C on the stacking fault energy (SFE) of an equiatomic CoCrNi medium entropy alloy. Results of computer modeling were compared to tensile deformation and electron microscopy data. Both N and C in solid solution increase the SFE of the face-centered cubic (FCC) alloy matrix at room temperature, with the former having a more significant effect by 240% for 0.5 at % N. Total energy calculations based on density functional theory (DFT) as well as thermodynamic modeling of the Gibbs free energy with the CALPHAD (CALculation of PHAse Diagrams) method reveal a stabilizing effect of N and C interstitials on the FCC lattice with respect to the hexagonal close-packed (HCP) CoCrNi-X (X: N, C) lattice. Scanning transmission electron microscopy (STEM) measurements of the width of dissociated ½<110> dislocations suggest that the SFE of CoCrNi increases from 22 to 42–44 mJ·m^−2^ after doping the alloy with 0.5 at. % interstitial N. The higher SFE reduces the nucleation rates of twins, leading to an increase in the critical stress required to trigger deformation twinning, an effect which can be used to design load-dependent strain hardening response.

## GRAPHICAL ABSTRACT

**Figure:**
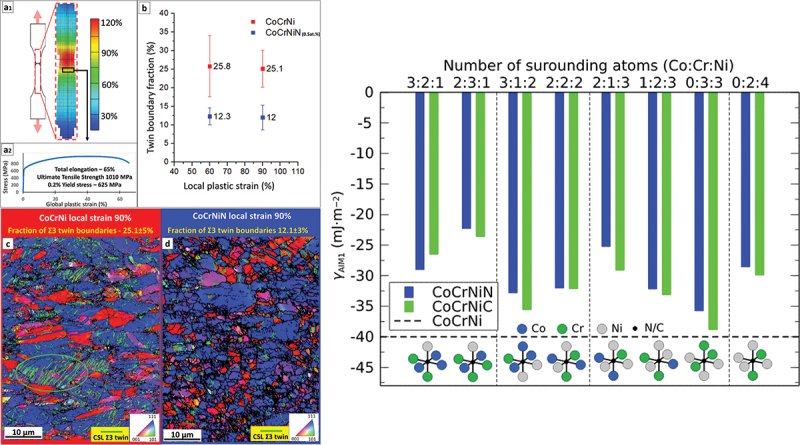

## 1. Introduction

Metals with face-centered cubic (FCC) crystal structures, such as Al alloys, Ni-base alloys or austenitic steels, play an important role in the industry due to their favorable combination of mechanical and physical properties [1,2]. In FCC metals, plastic deformation proceeds primarily through the multiplication and motion of *a*/2 <011> dislocations on {111} planes (*a* being the lattice parameter). The compact *a*/2 <011> dislocations dissociate into two Shockley partial dislocations of *a*/6 <112> type in FCC alloys with low stacking fault energies (SFEs). Low SFE alloys which are defined by SFE/*G*·*b* < 10^−3^ [3], where *G* is the shear modulus and *b* the magnitude of the Burger`s vector, are characterized by a large separation of the Shockley partials *d* of the order of *d*_edge_ >50*b* and *d*_screw_ >10*b* [3,4]. The two Shockley partials on the same {111} slip plane delimit a stacking fault (SF) ribbon [5]. The width of the ribbon is given by a balance between repelling forces caused by retraction of parallel components of two Shockley partials and the attractive force due to the SF [6]. The planar defect represents an intrinsic stacking fault (ISF) because it locally changes the {111} - ABCABC stacking sequence of the FCC matrix on one plane to the ABAB stacking of the hexagonal close-packed (HCP) phase [7]. The energy-balance of the crystal influenced by the SFE plays a significant role in the overall microstructural evolution of the material.

The force equilibrium between two Shockley partials is modified by external stresses. In materials with low SFE, Shockley partials can assume larger distances of up to 10–50*b* and trigger formation of deformation twins, enabling the twinning induced plasticity effect (TWIP) [8] resulting in enhanced strain-hardening rate. Despite significant differences between the twinning models, they all agree on certain general aspects: a) partial dislocation slip precedes deformation twinning, and b) twinning occurs after a twin nucleus is formed by the preceding reaction of dislocations that lead to specific configurations mostly at grain boundaries [9]. The interactions transpire between primary and secondary slip dislocations on either coplanar or non-coplanar slip systems [10,11]. The exact dislocation reaction and conditions at which a twin nucleus (embryo) forms are still under debate [12]. Various models and their characteristics are described elsewhere [12]. In materials with even lower SFE than TWIP alloys (<15 mJ·m^−2^), phase transformations from FCC to HCP, or even body centered cubic (BCC) and body centered tetragonal (BCT) phase regions [13,14] can occur. All these effects change the strain hardening behavior as the associated additional interface populations reduce the mean free path of the mobile dislocations, thus requiring higher loads to maintain plastic flow which increases strain hardening [8]. In some cases, even physical properties such as magnetism can be affected, particularly when it triggers the formation of paramagnetic BCT martensite [15–17]. Accordingly, the SFE is one of the main material parameters governing the microstructure and plastic deformation of the FCC metals [18–20]. Chemical compositions of the alloys can be designed in a way to target certain values of the SFE [20]. However, the experimental evaluation of the SFE values is demanding, due to its composition and temperature dependence [21]. On the other hand, methodologies based on CALPHAD (CALculation of PHAse Diagrams) thermodynamic modeling or *ab initio* calculations are available, which proved to be efficient in the prediction of SFEs [18,22,23].

If the FCC/HCP interface energy of the ISF and transformation strain energy [24,25] are neglected, the SFE can be approximated by the first-order axial Ising model (AIM1) [19,26]. In the model, the energy *γ*_AIM1_ proportional to the SFE is calculated as:

(1) $$\gamma_{AIM1}=2\left(E^{HCP}-E^{FCC}\right)/A_{111},$$

where *E*^*HCP*^ and *E*^*FCC*^ denote the energies of the individual HCP and FCC lattices, respectively, and *A*_111_ is the surface area of the {111} close packed plane calculated from the lattice parameter of the FCC structure *a*_*FCC*_. For the estimation of more realistic values of the SFE than those obtained by the AIM1, the direct difference of FCC-lattice energies with and without a stacking fault per surface area has to be used instead [19]. However, *γ*_AIM1_ values correlate very well with the results from the advanced model [22]. Therefore, the AIM1 provides a good approximation for estimating trends of the change in the SFE upon composition tuning, although it is not able to capture local effects such as mutual position of stacking fault and interstitial defect, which can also have an influence on the SFE [27–29].

*Ab initio* calculations based on density functional theory are usually employed to obtain input energies for Equation 1. This method provides the ground state energy of the lattice (or heat of formation) at 0 K and therefore the SFE values calculated from *ab initio* cover only the internal energy of the system. Various entropy effects (e.g. vibrational, magnetic, configurational, electronic, etc.) are often not included in such calculations, which makes the estimated SFE meaningful only for 0 K conditions. However, the values obtained by Equation 1 represent valuable information about the nature of the chemical effects influencing the SFE at an atomic level. In order to get insights also into compositional trends of the SFE at finite temperatures, the temperature-dependent Gibbs free energies have to be used within the AIM1 model. It is theoretically possible to obtain free energies covering all entropy contributions from *ab initio*, but it is computationally very demanding [30,31]. Instead of this, semi-empirical inputs to AIM1 within the certain temperature range can be estimated by CALPHAD thermodynamic modeling, in which parameter-fitted mean-field thermodynamic approximations of temperature-dependent Gibbs free energies of an alloy with desired composition are derived from experimentally estimated free energies of pure constituents of the alloy. They have the advantage of considering approximations to both the enthalpic and entropic portions of the free energy functions; hence, they are generally both composition- and temperature-sensitive but they have the disadvantage that some contributions are fitted from experimental data [32]. Although the theoretically estimated values of SFE are based on several approximations, they may still significantly contribute to the design of new materials with tailored stress-strain response, such as targeted for high strain-hardenability for energy absorption materials [12,19,31].

One such new material is the FCC equiatomic CoCrNi medium entropy alloy (MEA) which has an excellent combination of strength, ductility and longevity, when exposed to hydrogen or galvanic corrosion [33–35]. The MEAs are a group of materials usually composed of 3–4 elements in equiatomic or near equiatomic proportions that has received considerable attention due to its underlying novel alloy design concept [36,37]. The favorable strength-ductility combination of the CoCrNi MEA is at least partially related to its low SFE values of 18 ± 4 [38] or 22 ± 4 mJ·m^−2^ [39] at 293 K, and the associated TWIP effect.

A recent *ab initio* study conducted for the case of 0 K reported that the SFE of the CoCrNi solid solution can even reach negative values [40]. The negative SFE indicates an energetic preference for the HCP lattice over the FCC lattice at low temperatures like in the case of pure cobalt in its meta-stable FCC lattice structure, for which *ab initio* methods predict also negative SFE at 0K [31]. As the HCP Co is stable up to a critical temperature *T*^*FCC→HCP*^ at ~700 K, the metastable FCC Co exhibits negative SFE even at room temperature [41]. The correct temperature dependence of the SFE with positive values above *T*^*FCC→HCP*^ can be obtained from *ab initio* simulations only if all different contributions to the free energy are taken into account (vibrational, magnetic, electronic, volume expansion) [42].

Similarly to calculations for pure Co, Zhao et al. and later Niu et al. showed that the vibrational contribution (phonons) to the free energy stabilizes the FCC lattice of the CoCrNi over the HCP lattice and the SFE becomes positive above a critical temperature *T*^*FCC→HCP*^ [43,44]. Moreover, Ding et al. reported that the FCC structure of the CrCrNi can be stabilized even at low temperatures by varying the material’s chemical short range order (SRO) in a CoCrNi alloy, which results in the SFE at 0 K being in the range from −43 to 30 mJ·m^−2^ depending on local atomic arrangements [22].

We showed recently that the strength-ductility balance of the CoCrNi MEA can be further improved by interstitial alloying with C and N [45,46]. Interstitials in FCC solid solution significantly raise the critical resolved shear stress and, thus, the yield strength. This is associated with an increase in the elastic lattice strain calculated as a change in the lattice parameter *da* with the interstitial concentration *dx*: $\varepsilon_{E}=da/\left(a\cdot dx\right)$. In contrast to doping with C, which results in the precipitation of carbides during annealing [46], N does not form nitrides and remains dissolved in the FCC matrix even after recrystallization at 800–1000°C [45]. N also induces 72% higher lattice strain ($\varepsilon_{E}$ = 0.38 per at.%) than C ($\varepsilon_{E}$ = 0.22 per at.%) [47]. In addition to the change in yield strength, it was shown that interstitials lead to significant changes in the SFE of FCC metals, where austenitic steels and the FeCoCrMnNi alloy serve as examples [48–50].

In this study, we investigate the influence of interstitial N on the SFE of the CoCrNi MEA using both theory and experiments. We first study the influence using thermodynamic and *ab initio* calculations including also the effects of interstitial C for comparison. The results of the calculations are then cross-checked by experiments focused on the extent of deformation twinning and also by direct measurements of stacking fault widths using scanning transmission electron microscopy (STEM).

## 2. Methodology

We differentiate between the experimentally measured value of the SFE and the theoretical value of the energy *γ*_AIM1_ calculated within the AIM1 [19,26] approach for 0 K by *ab initio* calculations and for finite temperatures by semi-empirical thermodynamic modeling with the help of the CALPHAD method. *Ab initio* simulation was used to probe atomic-scale configurations of the interstitials at 0 K while CALPHAD modeling was used to evaluate the SFE’s temperature dependence. Differences between the results obtained by these two methodologies were also analyzed. Although the *γ*_AIM1_ values are not directly comparable with measured SFE values, they should be in qualitative agreement. Thus, the knowledge of *γ*_AIM1_ provides insights into the nature of the differences in the SFEs on an atomic level.

### 2.1. Ab initio *calculations*

The presented *ab initio* calculations at 0 K of *γ*_AIM1_ were performed using the Vienna Ab Initio Simulation Package (VASP) [51,52] in which the electron–ion interaction was described by projector augmented wave (PAW) potentials [53,54]. The electronic orbitals were expanded in terms of plane waves with a maximum kinetic energy of 450 eV considering explicitly the 3d^8^4s^1^ electrons as valence states for Co, 3d^5^4s^1^ for Cr, 3d^9^4s^1^ for Ni, and 2s^2^2p^3^ for N, and 2s^2^2p^2^ for C. We used the gradient-corrected exchange–correlation functional proposed by Perdew, Burke and Ernzerhof [55]. The Brillouin zone (BZ) of the computational cell was sampled using the 4×4×4 mesh centered around the Γ-point. The Methfessel-Paxton smearing method [56] with a 0.1 eV smearing width was applied during the integration over the BZ. The total energy was calculated with a convergence to 10^−6^ eV per the computational cell. In order to assess a magnetic contribution, our calculations were performed without and with spin-polarization, although the CoCrNi alloy exhibits a Curie temperature below 5 K [57]. Because we do not include any contribution to the free energy (e.g, vibrational, configurational or magnetic), the results of all *ab initio* calculations presented in this work do not consider any temperature effects and correspond to 0 K.

The equiatomic CoCrNi alloy was modeled based on the special quasi-random structures (SQS) approach [58]. To avoid the effects caused by SRO, we used large and fully disordered supercells with 216 atoms for both FCC and HCP structures. The supercells consisted of six {111} layers, each containing 36 atoms. The FCC supercell was generated using a Python tool [59] that optimizes pair correlation coefficients to form a statistically random solid solution. To overcome the very low symmetry of the model for solid solution describing HEA in comparison to the monoatomic FCC lattice, we employed a directional optimization of the randomly distributed atoms within the SQS supercell [60], which efficiently reduces the deviations between the symmetry-equivalent crystallographic directions. The HCP supercell was obtained from the FCC supercell by changing the stacking sequence of the close-packed planes from ‘ABCABC’ to ‘ABABAB’ while keeping the local arrangements of atoms in both structures as similar as possible. Atomic positions and lattice parameters were optimized with the external tool GADGET developed by Bucko et al. [61]. This application uses symmetry-adapted generalized coordinates and preserves the FCC or HCP geometry of the supercell during the relaxation process. The optimization was terminated when all forces acting on the atoms converged to within 10^−3^ eV·Å^−2^ and all relevant components of the stress tensor changed less than 0.1 GPa. For the Bader analysis of the calculated charge distribution, the software developed by Henkelman et al. [62,63] was used.

*Ab initio* calculations based on these settings for 0 K yielded FCC lattice parameters *a*_*FCC*_ = 0.3512 nm (*a*_*FCC*_ = 0.3523 nm when spin-polarization was considered) and HCP lattice parameters *a*_*HCP*_ = 0.2493 nm and *c*/*a* = 1.609 (*a*_*HCP*_ = 0.2500 nm and *c*/*a* = 1.613 for the spin polarization). These values are in good agreement with previously published *ab initio* data for 0 K [40,43]. The heat of formations $H_{f}^{0K}$ of various configurations were calculated with respect to the total energies of pure elements in their standard states, i.e. ferromagnetic HCP Co and FCC Ni and antiferromagnetic BCC Cr, which are 9.59 kJ·mol^−1^ and 8.94 kJ·mol^−1^ for the HCP and FCC structure, respectively. The positive $H_{f}^{0K}$indicates that the alloy is metastable at 0 K and has to be stabilized by configurational or vibrational entropy at finite temperatures. However, a similar value of 8.11 kJ·mol^−1^ at 0 K was recently reported for FeCoCrNi alloy [64]. The alloys doped by interstitials were modeled by adding one single C or N atom into the supercell, which resulted in molar concentrations of the doping interstitial elements of ~0.5%. Only octahedral sites in the supercells were considered based on previous results published for the FeCoCrMnNi alloy [18]. The total energy of the diamond and N_2_ molecule was considered for $H_{f}^{0K}$ calculations of alloys with interstitials.

### 2.2. Thermodynamic modeling

Phase equilibria of the CoCrNi, CoCrNiC, and CoCrNiN alloys at finite temperatures above room temperature were investigated by semi-empirical CALPHAD modeling using the Thermo-Calc software [65] and the TCHEA3.1 database developed on Scientific Group Thermodata Europe (SGTE) data for high and medium entropy alloys [66,67]. The CALPHAD method uses temperature-dependent Gibbs energies of all phases described in a given database to find the most stable phase for selected conditions by energy minimization. The CALPHAD databases are constructed based on measurements of different thermodynamic properties of simple systems (binary and ternary). Gibbs energies of individual phases are then fitted to these measurements using Redlich-Kister polynomials. Finally, the thermodynamic properties of compositionally more complex systems are calculated from these temperature-dependent fitted polynomials. The TCHEA 3.1 database includes all binary and ternary subsystems of the Co, Cr, Ni, C, N quinary systems, where almost all possible metastable and stable phases are considered. Due to the semi-empirical nature of CALPHAD modeling, the exact contributions of the entropic terms (configurational, magnetic and vibrational) cannot be distinguished, but are inherently included in the calculations.

The calculations were performed for the Co_33.3_Cr_33.3_Ni_33.3_, Co_33.2_Cr_33.2_Ni_33.2_N_0.5_, and Co_33.2_Cr_33.2_Ni_33.2_C_0.5_ compositions (in at. %). The CALPHAD framework is already adjusted for treating the N and C atoms as interstitials in both FCC and HCP CoCrNi lattices (as well as in other systems). The temperature dependencies of the Gibbs free energies were evaluated by the CALPHAD method for the single-phase HCP and FCC models at temperatures from 298 K to 1600 K. The room temperature is the lowest temperature at which the results based on the current SGTE database are meaningful [68,69].

The *γ*_AIM1_ values approximating the SFE at finite temperatures were estimated from these Gibbs free energies utilizing the AIM1 model. The surface area of the {111} close packed plane *A*_*111*_, see Equation 1, was calculated for the FCC lattice as $A_{111}\;=\left(a^{2}\times \sqrt{3}/2\times (N_{A}/2\right)$, yielding *A*_*111*_ = 31.86 × 10^3^ m^2^·mol^−1^ with an experimental FCC lattice constant of the CoCrNi a = 0.3568 nm [45] and Avogadro constant *N*_*A*_ *=* 6.022 × 10^23^. An increase in the lattice parameter by C or N is negligible (below 0.2%) for the presented calculations.

### 2.3. Preparation of alloys

The 25 × 60 × 60 mm^3^ ingots of the equiatomic *N*-free CoCrNi and N alloyed Co_33.2_Cr_33.2_Ni_33.2_N_0.5_ (CoCrNiN in what follows) MEAs were manufactured by vacuum induction melting with the chemical compositions given in Table 1. Pure metal feedstock in the form of pieces and lump of purity higher than 99.8% was used together with the FeCrN_2_ precursor alloy as a N source. As a consequence, the CoCrNiN alloy was enriched by about 0.7 at. % Fe. Plates with nominal dimensions of 10 × 25 × 60 mm^3^ were cut from the as-cast ingots by means of electric discharge machining (EDM FANUC Robocut, Japan) and hot-rolled at 1050°C. During the hot rolling, the slab thickness was reduced by 50% from 10 mm down to 5 mm. Hot-rolled plates were homogenized at 1200°C for 2 h in Ar atmosphere and water-quenched. Homogenized plates were subsequently cold-rolled with a 70% reduction in thickness (from 5 mm to ~1.5 mm) and then recrystallized in Ar protective atmosphere at 800°C for 30 min. and water quenched in order to obtain fine-grained microstructures. The treatment resulted in fully recovered, strain-free microstructures as reported elsewhere [45]. Water quenching after each step was intentionally used to prevent the formation of short range ordering effects, observed in the CoCrNi after slow furnace cooling in previous study [70].

**Table 1.** Chemical compositions of the as-cast CoCrNi and CoCrNiN alloys analyzed by inductively coupled plasma mass spectrometry.

Alloy		N	Co	Cr	Ni	Fe	O	S
CoCrNi	(wt. %)	0.005	35.2	30.7	34.0	0.06	0.012	0.0013
	(at. %)	0.02	33.7	33.4	32.7	0.06	0.04	0.0015
CoCrNi-N	(wt. %)	0.117	34.7	31.3	33.2	0.67	0.017	0.0019
	(at. %)	0.470	33.1	33.8	31.8	0.67	0.06	0.0025

### 2.4. Microstructural characterization

Samples for microstructure probing were ground with SiC paper and polished with 3 and 1 μm diamond pastes. A colloidal silica suspension (Struers OPS) was used for the final step to remove damaged surface layers. The microstructure was investigated by a Zeiss Ultra Plus scanning electron microscope (SEM, Zeiss, Germany) equipped with detectors for Electron Back Scattered Diffraction (EBSD) and Back-Scattered Electron Imaging (BSEI). At least three EBSD measurements were performed at each investigated sample area to cover a statistically relevant portion of the microstructure. The grain sizes of the materials were calculated according to ASTM E112 in HKL CHANNEL5 software excluding the twin boundaries and with the minimum grain boundary misorientation angle of 15°. STEM specimens were cut from tensile samples deformed to 3% of global strain. The cut pieces were mechanically thinned on SiC emery papers down to 0.1 mm. The 3 mm diameter discs were stamped from the thinned sheets and electrolytically polished in a twin jet polishing system (TenuPol5). The electrolyte consisted of 95% acetic and 5% perchloric acid. The optimum polishing conditions were obtained at 80–90 V, 0.2 mA, and a temperature between 12°C and 16°C. Further details on the preparation of the STEM samples can be found elsewhere [71]. STEM investigations were conducted using a JEOL JEM 2100 microscope (JEOL, USA) operated at 200 kV. We have combined tilting experiments, stereo pair, cube projection and weak beam techniques in order to provide information on crystallography and spatial distribution of defects [72–74].

### 2.5. Mechanical testing

Rectangular dog-bone shaped tensile specimens with a total length of 20 mm, gauge length of 4 mm, thickness of 1.5 mm and gauge width of 2 mm were machined from the recrystallized sheets. Tensile axes were oriented parallel to the former rolling direction. A Kammrath & Weiss (Kammrath & Weiss GmbH, Germany) tensile stage was used for the experiments performed at room temperature. The stage was operated in a displacement-control mode at an initial engineering strain rate of 1 × 10^−3^ s^−1^. The local strains were probed with the Aramis system (GOM GmbH) [75], and further details have been described elsewhere [45,46]. The deformed microstructures of these samples were further probed by EBSD.

For high-precision measurements at lower strains, a second tensile test was carried out for the CoCrNiN alloy. A dog-bone tensile sample was used with a 1.5 × 3 mm^2^ cross-section and a 14 mm gauge length with an extensometer attached directly to the sample. The tensile test was performed on a Z050 universal testing machine (Zwick/Roell GmbH, Germany) at an initial engineering strain rate of 1 × 10^−3^ s^−1^. STEM specimens were cut out from the sample deformed to 3% of global strain to analyze the deformed microstructures and measure the SFE values, as described above.

## 3. Results

### 3.1. Ab initio *calculations*

In agreement with previously published data on the fully disordered alloy [22], we found that the HCP structure is energetically more favorable at 0 K as compared to the FCC structure for both non-spin-polarized and spin-polarized calculations. The *γ*_AIM1_ calculated within the AIM1 model without spin polarization is equal to −40 mJ·m^−2^. This value falls into the range of the SFE reported by Ding et al. (from −43 to 30 mJ·m^−2^) [22] and is also comparable to other previous *ab initio* calculations of *γ*_AIM1_ (−26 mJ·m^−2^ [76] and −21 mJ·m^−2^ [43]), all performed for 0 K. The calculations with spin polarization provide *γ*_AIM1_ values equal to −42 mJ·m^−2^. Because both *γ*_AIM1_ values are very similar and magnetism has only a negligible effect on the energy approximated by the AIM1 model at 0 K [44], we will further discuss only the results obtained in the non-spin-polarized calculations.

**Figure 1.**
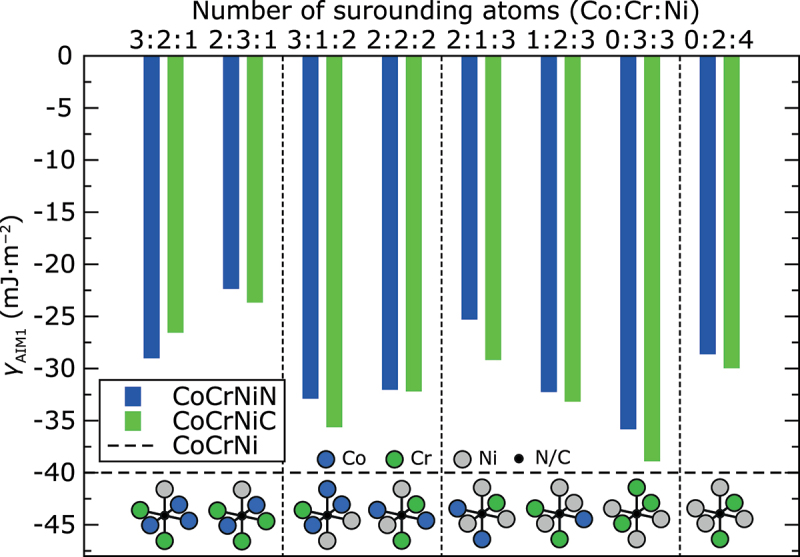
*Ab initio* γ_AIM1_ values of CoCrNiN andCoCrNiC alloys at 0 K calculated without spin-polarization for N and C atoms situated in different octahedral sites. The horizontal dashed line corresponds to the calculated γ_AIM1_ of the CoCrNi alloy without interstitials. The results are sorted in order of increasing number of Ni atoms and then increasing number of Cr atoms forming the octahedral site. Vertical thin dashed lines separate the results for octahedral sites with the same number of Ni atoms. Schematic representation of each octahedral site is shown in the bottom part of the figure.

A local chemical environment around interstitial atoms may alter the lattice formation energies. There are 24 configurationally different combinations of Co, Cr, and Ni atoms to form octahedral sites. Here, we calculated the heat of formation $H_{f}^{0K}$and *γ*_AIM1_ values at 0 K for supercells with N and C atoms situated in eight randomly chosen different types of octahedral sites. Figure 1 shows the *ab initio γ*_AIM1_ values for interstitials in each of the studied octahedral sites. In all cases, N and C additions increase the *γ*_AIM1_. Specifically, the C additions increase the *γ*_AIM1_ on average by 10 mJ·m^−2^ at 0 K, whereas N accounts for an average increase of *γ*_AIM1_ by about 8 mJ·m^−2^. However, in both cases, the increase in the *γ*_AIM1_ is nearly 25% as compared to the interstitial-free CoCrNi alloy.

**Figure 2.**
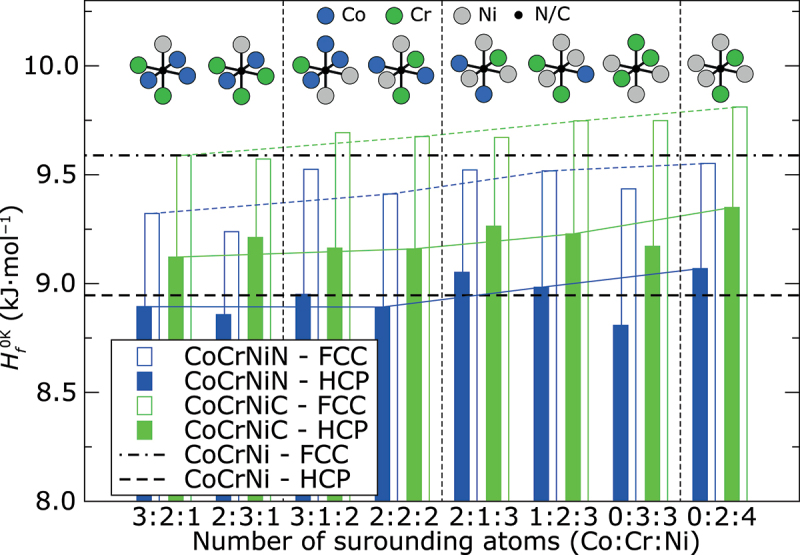
*Ab initio* heat of formation $H_{f}^{0K}$ of CoCrNiN and CoCrNiC alloys at 0 K calculated without spin-polarization for N and C atoms situated in the same octahedral sites as on Figure 1. The black horizontal lines correspond to the calculated $H_{f}^{0K}$ of the interstitial-free CoCrNi alloy with FCC and HCP structures. The results are sorted in the same order as in Figure 1. Thin color lines join the results for octahedral sites formed by the same number of Cr atoms (2 Cr atoms). Vertical thin dashed lines separate the results for octahedral sites with the same number of Ni atoms. Schematic representation of each octahedral site is shown in the upper part of the figure. Atomic configurations are identical as in Figure 1.

The calculated values for the heat of formation$H_{f}^{0K}\;$ pertaining to the identical atomic configurations as in Figure 1 are summarized in Figure 2. These values represent the relative stability of the alloy with respect to the pure constituents in their ground states at 0 K. They can be also used for estimation of preferred position for C or N interstitials as the preferred sites should exhibit the lowest heat of formation. Results in Figure 2 reveal that all studied sites in the FCC structure occupied by N interstitial decrease the heat of formation compared to *N*-free FCC structures. In the HCP structure, the decrease of $H_{f}^{0K}$ by N can be found only in some cases. On the other hand, interstitial C always significantly increases $H_{f}^{0K}$in HCP structure as well as in most cases in the FCC structure. This finding corresponds very well to the experimentally observed better solubility of N in the CoCrNi alloy compared to C [46]. The lowest $H_{f}^{0K}$ in the FCC structure is reached when N and C atoms occupy an octahedral site surrounded by two Co atoms, three Cr atoms and one Ni atom (see empty bars denoted as 2:3:1 in Figure 2). This configuration also exhibits the highest *γ*_AIM1_ from all calculated configurations: −24 mJ·m^−2^ for the CoCrNiN alloy and −22 mJ·m^−2^ for the CoCrNiC alloy. Based on our calculations, the N atom in the same octahedral site exhibits very low $H_{f}^{0K}$ also in the HCP structure, lower by about 88 J·mol^−1^ than in the alloy without interstitial blending. However, the lowest $H_{f}^{0K}$ in the HCP structure was found for N surrounded by three Cr atoms and three Ni atoms, lower by 138 J·mol^−1^ compared to the alloy without interstitial. On the other hand, the highest $H_{f}^{0K}$ was always found for interstitials surrounded by two Cr atoms and four Ni atoms. Comparison of results for octahedral sites formed by equal number of Cr atoms (2 Cr atoms) suggests that $H_{f}^{0K}$ will increase with increasing number of Ni atoms surrounding the interstitial (see thin color lines in Figure 2). It is also possible to recognize the trend that $H_{f}^{0K}$ of the alloy with N interstitial decreases with increasing number of surrounding Cr atoms. The same trend is not so significant for C interstitial (compare results on Figure 2 in each section separated by vertical dashed lines), for which the site preference seems to be less sensitive to the surrounding atoms because the $H_{f}^{0K}$ values for different configurations vary much less compared to the $H_{f}^{0K}$ values of N interstitials. However, these results reveal that the interstitial atom rather prefers octahedral sites surrounded by more Cr atoms and less Ni atoms in both structures.

Such a trend could be explained by Pauling electronegativities and analysis of charge transfer between interstitial atom and atoms forming the octahedral sites. We employed Bader method [77] for this task which decompose the ab initio charge density of the whole supercell into the atomic contributions based on so-called Bader volumes. The faces of each Bader volume are located at the minimum of charge density between neighboring atoms. Therefore, the Bader volumes completely fill the volume of the supercell. We performed the analysis of charge redistribution for interstitials in two octahedral sites: for the most preferred site surrounded by 2 Co, 3 Cr and 1 Ni (2:3:1 configuration) and for site surrounded by 1 Co, 2 Cr and 3 Ni (1:2:3 configuration) with relatively high $H_{f}^{0K}$. For the alloy without interstitial the Bader method shows average charge transfer of 0.4 electrons from each Cr atom which is almost equally distributed to Ni and Co atoms. This charge transfer corresponds to higher Puling electronegativity of Ni and Co atoms (1.91 and 1.88, respectively) with respect to Cr atoms (1.66) and is nearly the same in FCC and HCP structure.

**Figure 3.**
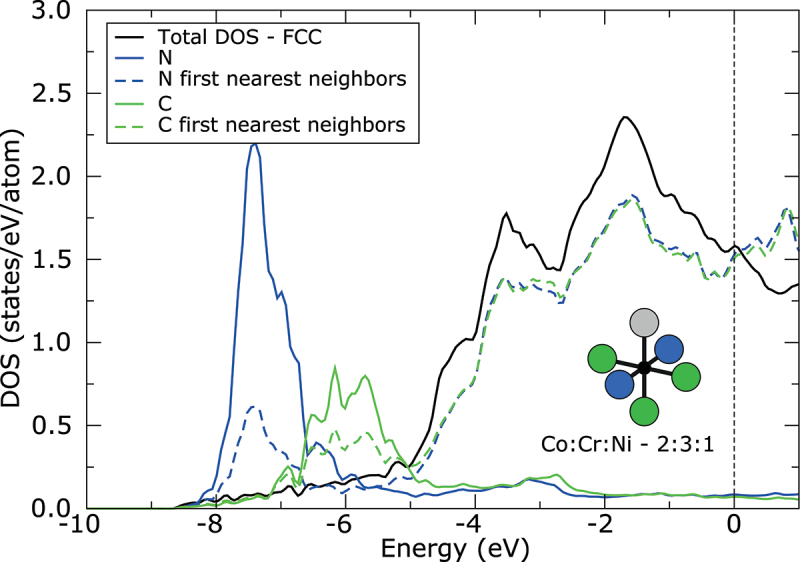
Density of states (DOS) of CoCrNiN and CoCrNiC alloys with FCC structure and with interstitials in octahedral site formed by 2 Co, 3 Cr and 1 Ni atom calculated without spin polarization. Black solid line corresponds to the total DOS per atom. The total DOS is nearly identical for CoCrNiN and CoCrNiC alloys. Blue and green solid lines correspond to the DOS of N and C interstitial, respectively, whereas blue and green dashed lines correspond to the average DOS of first nearest neighbors around interstitial, which form the octahedral site. The zero energy corresponds to the Fermi level.

Because both N and C interstitials exhibit high electronegativity of 3.04 and 2.55, respectively, large amount of charge is transferred from 3d metal atoms to the interstitial atom. As can be seen from density of states (DOS) displayed in Figure 3, the interaction between s electronic states of metallic atoms and p electronic states of interstitial atoms is realized far below the Fermi level (approximately −7.5 eV in case of N and −6 eV in case of C). Only the results for FCC structure and interstitials in 2:3:1 configuration are shown in Figure 3, due to very small differences in DOS between different octahedral sites and even between FCC and HCP structure. The charge on N interstitials increases approximately about 1.2 electrons with respect to neutral N atom. The charge transfer to C interstitials is slightly lower, due to its lower electronegativity. The charge on C increases approximately about 1 electron. However, the charge transfer is slightly larger, about 0.1 electrons, for both interstitials in the more stable configuration of octahedral site (2:3:1 configuration) with more Cr atoms forming the site. Surprisingly, the lowest charge transfer, between 0.11 and 0.17 electrons, was found from Cr atoms to the interstitial, although the Cr exhibits lower electronegativity than Ni or Co. On the other hand, the charge transfer from Co and Ni atoms to the interstitial is higher, between 0.16 and 0.23 electrons. It is explained by the fact that Cr atoms already provided their electrons to Co and Ni atoms. Therefore, the interstitials prefer to be surrounded by more Cr atoms exhibiting lower electronegativity, which can provide electrons for bonding easily. Moreover, the stability of each configuration can be further strongly influenced by second nearest neighbors around interstitial, which will affect the electronic structure of the first nearest neighbors and their ability to provide electrons for bonding. However, the deep analysis of second nearest neighbors is beyond the scope of this study.

### 3.2. Thermodynamic modeling

**Figure 4.**
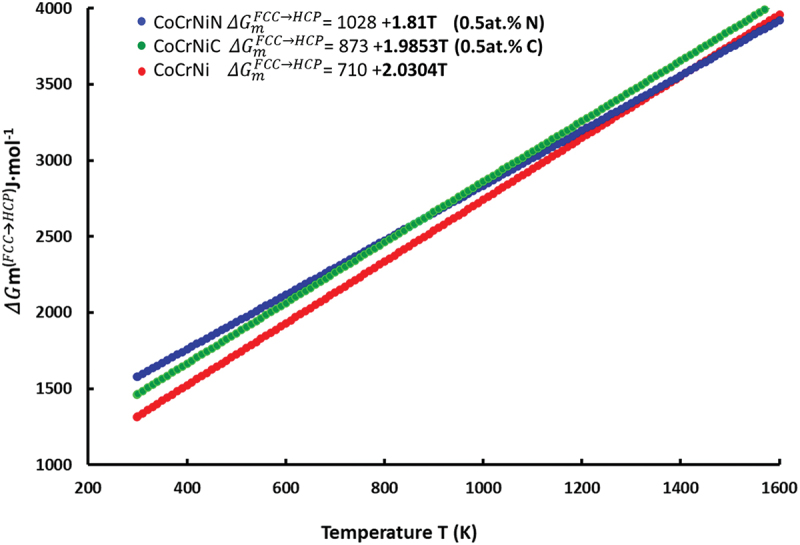
CALPHAD results showing the differences between the Gibbs free energies of the HCP phase and the FCC matrix phase $\Delta G_{m}^{FCC\to HCP}$. The relative thermodynamic stability of the FCC phase increases with temperature, with N alloying up to ~1400K and with C alloying beyond the studied temperature range.

From a thermodynamic point of view, the formation of ISF generates HCP stacking layers within the FCC matrix. Therefore, differences in Gibbs free energies of corresponding FCC and HCP lattices control the SFE values at finite temperatures. The semi-empirical CALPHAD method predicts the phase equilibria based on molar Gibbs free energies $G_{m}$ (J·mol^−1^) of the phases which may potentially form in the CoCrNi, CoCrNiN and CoCrNiC alloys. In general, an increase in the phase stability of any phase requires a decrease in the $G_{m}$ value (becoming more negative). The calculations were performed for compositions in a single-phase state (either FCC or HCP). The results of the calculations for a temperature of 298 K, at which the SEM, TEM and tensile tests were carried out, are presented in Table 2. The addition of N and C to the CoCrNi alters the Gibbs energy values calculated for both the FCC ($G_{m}^{FCC}$) and HCP ($G_{m}^{HCP}$) lattices. However, the most important value is the difference between the energy of the HCP phase regions created by the stacking fault and the adjacent intact FCC matrix phase $\Delta G_{m}^{FCC\to HCP}=G_{m}^{HCP}-G_{m}^{FCC}$. The temperature dependence of the $\Delta G_{m}^{FCC\to HCP}$ for all materials studied here is presented in Figure 4, including the data fitting line. The $\Delta G_{m}^{FCC\to HCP}$values at 293 K are 1315, 1578 and 1463 J·mol^−1^ for CoCrNi, CoCrNiN and CoCrNiC, respectively. The $\Delta G_{m}^{FCC\to HCP}$ values thus increase with N and C alloying as compared to the interstitial-free CoCrNi reference material. We note that N additions yield the highest increase. However, the CoCrNiN shows a lower value of the $\Delta G_{m}^{FCC\to HCP}$ slope (1.81) as a function of temperature *T* relative to the slope of CoCrNiC (1.98). Consequently, the $\Delta G_{m}^{FCC\to HCP}$ values of the CoCrNiC are increasing faster compared to the CoCrNiN, taking a lead at temperatures higher than ~850 K. The implications are discussed in Section 4. We did not extrapolate the results of thermodynamic modeling to 0 K even though it would allow a direct comparison with the results of the *ab initio* calculations. This is because changes in the real $G_{m}^{FCC}$ and $G_{m}^{HCP}$ with temperature below 298 K show strong non-linearities, which may cause faulty results concerning the $\Delta G_{m}^{FCC\to HCP}$. These non-linearities mainly arise from vibrational contribution to the free energy, as was pointed out by Zhao et al. and Niu et al. for the interstitial free CoCrNi alloy [43,44].

**Table 2.** Gibbs energies of the HCP stacking fault phase and the FCC matrix phase calculated by the CALPHAD method for 298 K. Gibbs free energy differences represent the relative stability of the two lattices in the given system.

CoCrNi (J·mol^−1^)			CoCrNiN (J·mol^−1^)			CoCrNiC (J·mol^−1^)		
$G_{m}^{FCC}$ phase	$G_{m}^{HCP}$ phase	$\Delta G_{m}^{FCC\to HCP}$	$G_{m}^{FCC}$ phase	$G_{m}^{HCP}$ phase	$\Delta G_{m}^{FCC\to HCP}$	$G_{m}^{FCC}$ phase	$G_{m}^{HCP}$ phase	$\Delta G_{m}^{FCC\to HCP}$
−7078	−5763	1315	−7533	−5955	1578	−7073	−5611	1463

Finally, the *γ*_AIM1_ is calculated by inserting the $\Delta G_{m}^{FCC\to HCP}$ values to Equation 1. The *γ*_AIM1_ values predicted by the Thermo-Calc for the CoCrNi, CoCrNiC and CoCrNiN alloys at 298 K are 41.3, 43.6, and 46.8 J·mol^−1^, respectively.

### 3.3. Microstructures after tensile deformation

**Figure 5.**
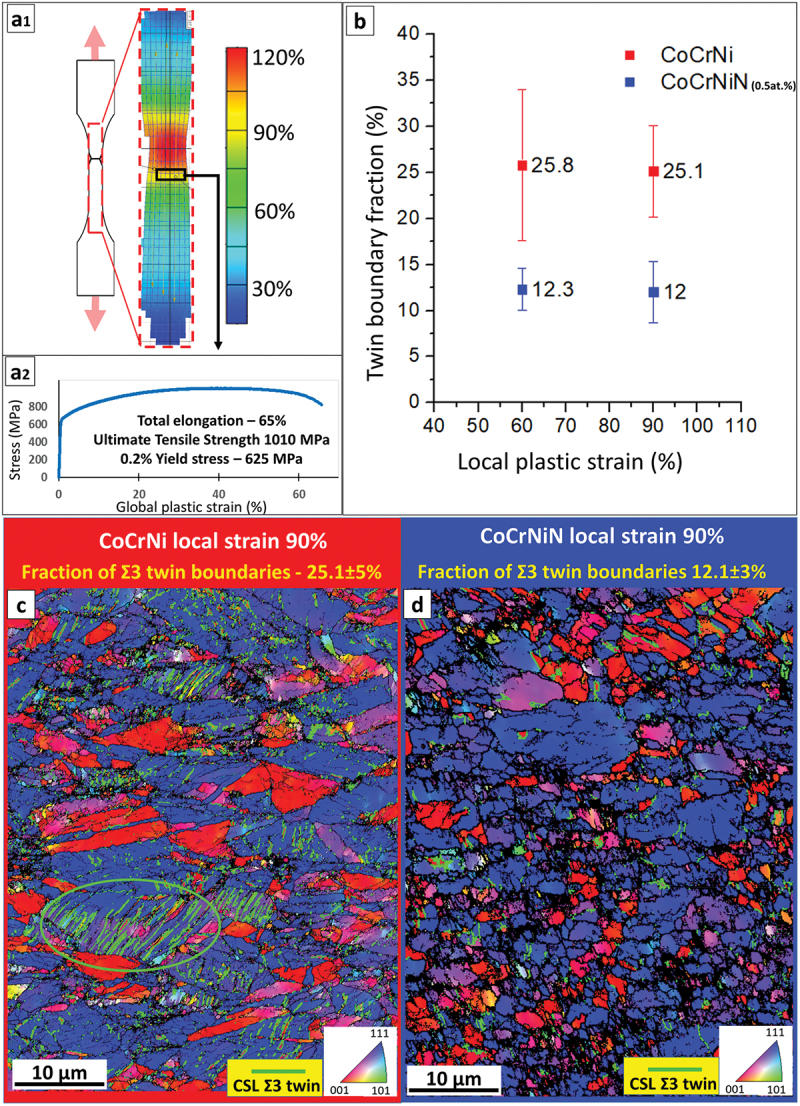
Microstructure evolution upon tensile deformation of CoCrNi and CoCrNiN alloys. (a) Results of the tensile testing showing; (a_1_) distribution of local strains in the CoCrNiN tensile sample just before the rupture event as determined by the DIC method with the red area corresponds to the necking region; (a_2_) corresponding tensile curve and global mechanical properties of the CoCrNiN sample from which local strain distribution is displayed. (b) Fractions of Ʃ3 twin boundaries in sample areas with engineering local strains of 60% and 90%, representing average from three measurements. (c) EBSD inverse pole figure (IPF) map of the CoCrNi alloy at 90% local strain. Twin boundaries in the elliptical region are marked by light-green lines. (d) EBSD IPF map of the CoCrNiN alloy at 90% local strain.

The CoCrNi and N alloyed CoCrNiN MEAs in their recrystallized state exhibited single-phase fine-grained FCC microstructures with comparable average grain sizes of 3.3 and 3.8 μm, respectively, according to EBSD analysis. Both recrystallized alloys retained a relatively high fraction of ~55–60% of Ʃ3 annealing twin boundaries. More details on the microstructures and global mechanical properties of both alloys can be found in our recent publications [45,46]. Figure 5 shows the analysis of local strains and microstructures of both alloys upon tensile testing. Local strains evaluated by digital image correlation (DIC) for the CoCrNiN sample are shown in Figure 5(a_1_), with the tensile curve and global mechanical properties presented in Figure 5(a_2_). Subsequent EBSD analysis (Figures 5(c,d)) focused on locations with relatively large local strains of 60% and 90% (the rectangular area highlighted in Figure 5(a)). The analysis aimed at a quantification of deformation twinning, particularly at a percentage of *deformation* twin boundaries in the overall boundary system. A reason for the particular selection of highly deformed areas is to avoid the contribution of the *annealing* twin boundaries, which, in the areas strained less than 60%, represent a large portion of the twin boundary ensemble. On the contrary, in regions with high local strains exceeding 60%, where the deformation twinning substantially contributes to the plasticity of the alloys, the portion of the original annealing twin boundaries becomes negligible. Therefore, the twin boundary fractions presented in Figure 5(b) correspond almost exclusively to *deformation* twins caused by plastic strain and not to *annealing* twins formed during recrystallization of the alloys. Both the deformed CoCrNi and CoCrNiN materials show a typical double-fibre <111> and <100> texture (Figure 5(c,d)). The fractions of deformation twin boundaries in the CoCrNiN material are in average lower by about 50% as compared to that in the CoCrNi MEA at identical local strain levels (Figure 5(b)). The lower tendency to form deformation twins in the *N*-doped CoCrNiN alloy suggests that its SFE is higher than that of the base CoCrNi alloy, as discussed later. A complete picture concerning the deformation twinning and its contribution to the total plastic strain in both alloys cannot be obtained only on the basis of the EBSD data. Most of the deformation twins have dimensions in the range from units to few tens of nm thus below the resolution limit of the EBSD method, which is able to properly resolve twins with thickness roughly over 50–100 nm (with current conditions and used experimental setup). This is visible from chunky twin boundaries in Figure 5(c,d) in addition to the line segments, pertaining to areas containing large number of very fine twins. Therefore, a more detailed investigation using STEM techniques was performed to directly assess the width of ISFs and to provide quantitative estimates of SFEs.

### 3.4. STEM results

**Figure 6.**
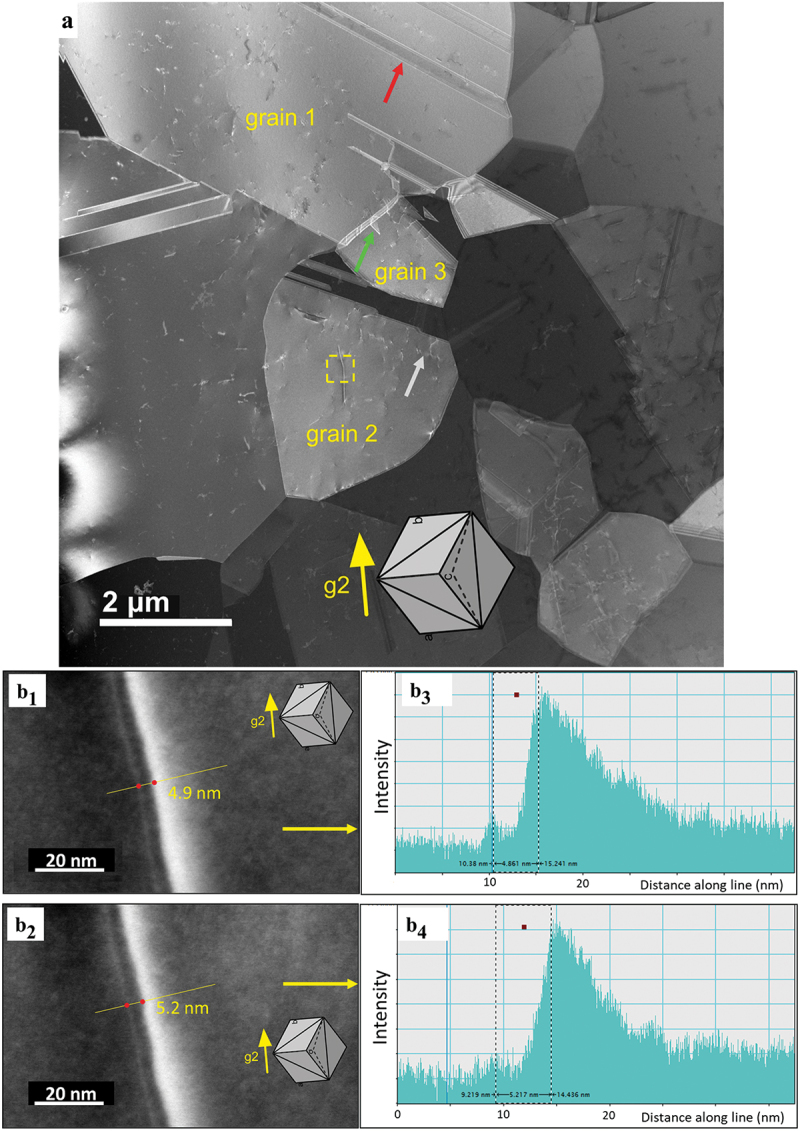
STEM images of the microstructure formed in the CoCrNiN MEA upon deformation to 3% tensile strain. (a) Overview HAADF-STEM image showing FCC grains marked as 1, 2 and 3. The operating diffraction condition in grain 2 was **g2** = ($\overset{ˉ}{2}$20). The annealing twin boundary, stacking fault and planar dislocation slip are marked by yellow, green and grey arrows, respectively. The yellow rectangle in grain 2 highlights the area with a dislocation dissociated into Shockley partials, which is displayed in detail in part b of the figure. (b) Contrast of the dissociated dislocation allows the experimental estimation of the dissociation distance between the two partial dislocations. The corresponding stacking fault width changes between 4.9–5.2 nm. The orientation of the grains is represented by the cube in the inset.

A typical microstructure of the CoCrNiN alloy deformed to 3% global tensile strain is documented in an overview STEM micrograph in Figure 6(a). The microstructure is composed exclusively of the FCC grains with annealing twins formed during recrystallization. One annealing twin is marked by a red arrow in grain 1. As compared to the recrystallized state, the dislocation density increased as a result of the 3% global tensile strain accumulated in the sample. The dislocations are often arranged in planar groups, as indicated by the grey arrow in grain 2 which indicates the {111}<110> planar slip reported recently [21]. The green arrow in Figure 6(a) marks the extended stacking fault nucleated from the grain boundary. Our tilting experiments allow us to conclude that the long dissociated dislocation, which crosses the yellow dashed rectangle in grain 2, shown in Figure 6(a), is located in the (11$\overset{ˉ}{1}$) crystallographic plane. A corresponding lattice of the grain 2 projected in line with the STEM foil tilting position is represented by the cube in the inset of Figure 6(a) [78]. This cube projection method reveals that the (11$\overset{ˉ}{1}$) crystallographic plane in grain 2 is almost parallel to the plane of the figure. The STEM contrast presented in Figure 6(b_1_,b_2_) further suggests that the dislocation line direction is close to the [$\overset{ˉ}{1}$10] crystallographic direction and that the dislocation is close to the 60° orientation, the Burgers vector being either ± a/2 [0$\overset{ˉ}{1}\overset{ˉ}{1}$] or ± a/2 [101], where *a* is the lattice parameter a = 0.3565 nm from XRD [46]. Therefore, there are two scenarios of how this 60° dislocation can dissociate into Shockley partials: (i) either according to a reaction ± a/2 [0$\overset{ˉ}{1}\overset{ˉ}{1}$] = a/6 [1$\overset{ˉ}{2}\overset{ˉ}{1}$] + a/6 [$\overset{ˉ}{1}\overset{ˉ}{1}\overset{ˉ}{2}$] or (ii) following a reaction ±a/2 [101] = a/6 [112] + a/6 [$\overset{ˉ}{2}\overset{ˉ}{1}\overset{ˉ}{1}$]. The experimental dissociation distances between the indicated Shockley partials (the ISF widths *w*) were analyzed in detail using tools of the DigitalMicrograph® software. The results presented in Figure 5(b_1_–b_4_) indicate that the experimental widths fall in the range *w* = 4.9–5.2 nm. Based on these experimental data, the SFE can be calculated using Equation 2 [79]:

(2) $$\;SFE=\frac{\;G}{2\;\pi w}\left[\left(\;b_{2}\;\cdot {\;\xi }_{2}\right)\left({\;b}_{3}\;\cdot {\;\xi }_{3}\right)+\frac{\left({\;b}_{2}\times {\;\xi }_{2}\right)\left({\;b}_{3}\times {\;\xi }_{3}\right)}{1-\;\nu }\right]$$

where the shear modulus *G* = 90 GPa and the Poisson ration *ν* = 0.31 represent the elasticity parameters reported for CoCrNi [80]. This selection of *G* and *ν* is guided by our earlier results, which provided evidence showing that interstitial alloying with 0.5 at. % N does not significantly affect the elastic properties of the CoCrNi MEA [46]. The variables *b*_*2*_,*b*_*3*_ and *ξ*_*2*_, *ξ*_*3*_ correspond to Burgers vectors and line directions of leading and trailing Shockey partials introduced in the two dissociation scenarios. The *b*_*2*_, *b*_*3*_ and *ξ*_*2*_, *ξ*_*3*_ data of both dissociated scenarios were substituted into Equation 2 together with the experimental dissociation widths *w*, yielding the SFE value of the CoCrNiN material in a range 42–44 mJ·m^−2^ at room temperature.

**Figure 7.**
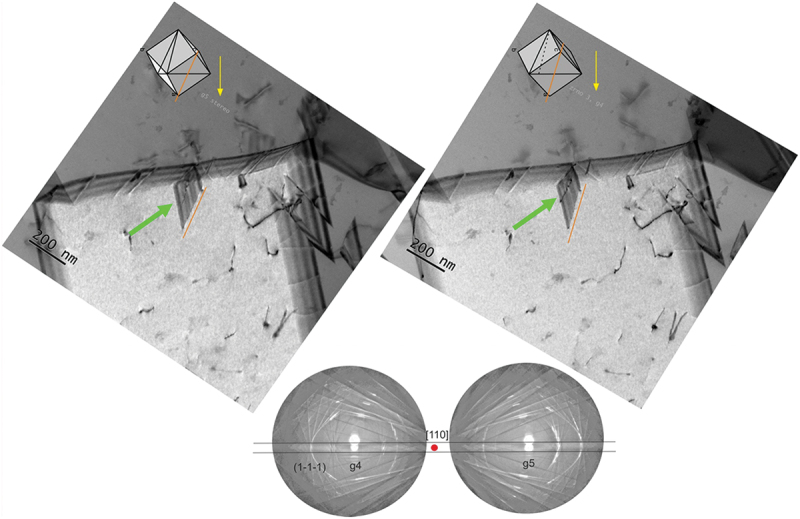
STEM bright field (BF) stereo pair documenting a nucleation of a fault (green arrow) from the grain boundary in the grain 3 marked in Figure 5(a). The diffraction condition in the grain 3 was **g4** = **g5** = (1$\overset{ˉ}{1}\overset{ˉ}{1}$). The orientation of the grains is represented by the cubes in the inset.

Extensive deformation twinning was not observed in the microstructure of the CoCrNiN MEA after 3% plastic tensile strain. Nevertheless, the STEM stereopair in Figure 7 documents an interesting event where an ISF or a deformation twin nucleus grows from a grain boundary into the interior of the grain 3. We note that grain 3 was marked in the overview image shown in Figure 6(a). The 3D perception of the microstructure provided by the stereopair [74] combined with the cube projection method [78] allows the crystallographic arrangement of the embryonic fault/deformation twin to be described. The two methods suggest that the fault/deformation twin moves parallel to the (111) crystallographic plane and the leading edge is parallel to the [10$\overset{ˉ}{1}$] crystallographic direction. Therefore, the ISF/deformation twins can still nucleate and grow in the CoCrNiN alloy and a minimum tensile strain of 3% is likely necessary to activate these deformation mechanisms.

## 4. Discussion

### 4.1. The increase in the SFE by interstitial N and C

A correlation between SFE values and stability of the competing FCC and HCP lattices is characterized by the value of *γ*_AIM1_ given by Equation 1 . Thus, the increase (decrease) in *γ*_AIM1_ indicates an increase (decrease) in SFE. The molar total (or Gibbs) energy difference $\Delta G_{m}^{FCC\to HCP}=G_{m}^{HCP}-G_{m}^{FCC}$ is the variable that most sensitively reflects the doping of the alloy by N and C. The results obtained with the help of the semi-empirical CALPHAD method suggest that at room temperature, the value of $G_{m}^{FCC\to HCP}$ increases upon the addition of N and C, which, according to Equation 1, should lead to an increase in the *γ*_AIM1_ value. This finding is in-line with the experimental results, which show the identical trend of increasing SFE values due to the doping by interstitial N and C [47]. Table 2 also documents that N causes a slightly higher increase in the $G_{m}^{FCC\to HCP}$ values compared to C, and, therefore, a larger (7%) increase in the *γ*_AIM1_. This trend is supported by the experiments even though the quantitative experimental effects are much larger. A doping by 0.5 at. % of N increases the SFE value by 21 mJ·m^−2^ whereas 0.75 at. % of C increases the SFE by only 7 mJ·m^−2^ [47], indicating that N is significantly (240%) more effective. However, a discrepancy between the *γ*_AIM1_ calculated at room temperature for the interstitial-free alloy CoCrNi (41.3 mJ·m^−2^) and the experimentally estimated value of SFE = 22 mJ·m^−2^ [39] indicates that the thermodynamic predictions based on the AIM1 model should be considered only on a qualitative basis.

The *ab initio* results indicate that additions of N and C to the CoCrNi base alloy lead to an increased relative stability of the FCC phase with respect to the HCP phase even at 0 K. Therefore, the *ab initio* calculations also suggest that the SFE of the CoCrNiN and CoCrNiC alloys may increase upon the addition of N or C. Although we did not consider the coherent FCC–HCP interfacial energy, the transformation strain energy or potentially occurring SRO effects [22], our theoretical prediction is in qualitative agreement with the experimental data. We obtained a negative *γ*_AIM1_ value, suggesting that the HCP phase exhibits a lower energy than the FCC phase at low temperatures, while interstitial N and C stabilize the later phase. We assume that the stabilization effect of interstitials on the FCC phase can also be expected for models that include SRO effects, where the SFE is positive and will increase as well. Moreover, in fully disordered alloys, the addition of N and C results in a less negative SFE, which will subsequently decrease the critical temperature *T*^*FCC→HCP*^ reported by Zhao et al. [43] at which the SFE becomes positive.

However, while the experiments and the semi-empirical CALPHAD predictions agree on the more significant increase in the SFE due to the doping by N at room temperature, the *ab initio* calculations suggest that C may cause a more significant effect at 0 K. It should also be pointed out that the *ab initio* calculations show relatively small differences in average *γ*_AIM1_ values between C and N alloying. In addition to the SFE values, *ab initio* results also rationalize higher solubility of N doped FCC CoCrNi phase compared to the C doped phase observed in the experiments [45,46], exhibited by lowering of heat of formation$H_{f}^{0K}\;$of all octahedral configurations by N.

### 4.2. Increased twinning stresses caused by interstitials

As explained in the introduction, a consensus on the detailed mechanism of the onset of deformation twinning is largely missing [6,12]. However, models agree that twin embryos at grain boundaries grow due to localized stress concentrations, exerting shear stress on the leading partials at the tip of twin embryos [10,11]. The stresses required for these mechanisms to occur are indirectly related to the grain size of the material, resulting in an apparent grain-size dependence of the twinning stress. The mechanistic reasons for this phenomenological dependence may be related to the same effects causing grain-boundary strengthening. For instance, higher required stress for dislocation motion with decreasing grain size can be related to higher dislocation density due to larger net grain surface area or increasing back-stress on dislocation sources by the dislocation pile-ups against boundaries due to a smaller grain diameter [81].

The twinning theories formulated for instance by the Miura–Takamura–Narita (MTN) model [82] and also by the model of Byun [6], predict that the shear stress to initiate mechanical twinning (separate partials) is proportional to the energy of the ISF. The MTN model showed the best fit to our experimental data as well as to similar low SFE MEAs [83] and austenitic steels [84]. The true tensile stress for the onset of twinning *σ*_*0T*_ (*T* in *σ*_*0T*_ stands for twinning) is calculated as [82]:

(3) $$\sigma_{0T}=M\left(\frac{SFE}{2b}\right)$$

Here, *M* is the Taylor factor for randomly textured FCC metals equal to 3.06 [85], and *b* is the magnitude of the Burgers vector of the Shockley partial dislocation. Equation 3 yields as value for the tensile twinning stress *σ*_*0T*_ = 231 MPa for the CoCrNi monocrystal with a SFE of 22 ± 4 mJ·m^−2^ [39] at room temperature. This value is almost identical to the experimentally observed twinning stress *σ*_*0TE*_ = *Mτ*_*OTE*_ = 239 MPa, where a shear twinning stress *τ*_*0T*_ = 78 MPa was obtained from a mechanical test of CoCrNi single-crystals [86] measured at room temperature (293 K).

For comparison, we show the results of calculations using twinning models proposed by different authors. The Mahajan – Chin model calculates the tensile twinning stress *σ*_*0T*_ as [8,9]

(4) $$\sigma_{0T}=M\left(\frac{SFE}{3b}+\;\frac{3Gb}{L_{0}}\right)$$

Here, $L_{0}$ is the width of the twin embryo, which is currently not measured for the CoCrNi alloy and presents the largest issue of this model. Other authors usually use for their calculations $L_{0}$ = 260 nm, originally utilized for Mn TWIP steels [8]. Thus, we obtain *σ*_*0T*_ = 614 MPa for single-crystal CoCrNi, which is too large compared to the available experimental value (239 MPa).

Another model proposed by Meyers-Vöhringer-Lubarda [87] estimates the twinning stress *σ*_*0T*_ as:

(5) $$\sigma_{0T}=\left(K2\;\sqrt{\frac{SFE}{Gb}}\right)$$

where the experimentally estimated constant K2 = 6 GPa gives *σ*_*0T*_ = 504 MPa and shows the second best fit to the experimentally determined single-crystal values (239 MPa).

Lastly, the model proposed by Byun uses an empirically estimated calculation of the twinning stress *σ*_*0T*_ [6]:

(6) $$\sigma_{0T}=\left(6.14\;\frac{SFE}{b}\right)$$

This model gives a value of *σ*_*0T*_ = 919 MPa, a large overestimation, which is not surprising owing to its purely empirical nature. *Ab initio* derived tensile twinning stresses of *σ*_*0T*_ = 890 MPa calculated from shear twinning stress τ_0T_ = 291 MPa [39] by *σ*_*0T*_ = *M*τ_*0T*_ also produce a large overestimation compared to the single-crystal result *σ*_*0T*_ = 239 MPa.

The true tensile stress for the onset of twinning in the CoCrNiN can be estimated using the SFE of 42–44 mJ·m^−2^ evaluated from our STEM data (see Section 3.4 STEM results). This SFE value can be substituted into Equation (3), yielding *σ*_*0T*_ = 452 MPa for the CoCrNiN which is 96% higher compared to *σ*_*0T*_ = 231 MPa calculated for CoCrNi. All these calculations are consistent with our EBSD measurements which confirmed a lower fraction of deformation twin boundaries (by about 50%) in the fine-grained CoCrNiN alloy compared to the CoCrNi reference material. A lower fraction of deformation twins also explains decreased strain hardening rates of the CoCrNiN alloy compared to CoCrNi with similar grain size [45]. Similarly, a decreased twin boundary fraction was recently also reported for the CoCrNi alloy doped by interstitial C [24,31].

## 5. Conclusions

We investigated the influence of interstitial N and C on the SFE values of the CoCrNi MEA using thermodynamic/CALPHAD modeling, *ab initio* calculations, and electron microscopy. The main conclusions are:

- Semi-empirical CALPHAD modeling, employing the AIM1 approach, predicted an increase in the SFE in the N and C doped CoCrNi alloy.
- Compared to the CoCrNiC alloy doped by C, N as solid solution in the CoCrNiN alloy causes a larger increase of the SFE, as revealed by experiments.
- *Ab initio* calculations predicted an increase in relative stability of the FCC lattice with respect to the HCP lattice at 0 K, which is associated with doping the CoCrNi alloy by interstitial N. These calculations thus support the results of the STEM experiments and thermodynamic modeling concerning the changes in the SFE due to interstitial doping.
- *Ab initio* calculations also predict higher solubility of N in the FCC phase of CoCrNi, compared to C, supported by experiments.
- STEM analysis of the dissociated ½<110> dislocations suggest that the CoCrNiN alloy doped by 0.5 at.% of interstitial N has the SFE value in the range of 42–44 mJ·m^−2^. This represents a significant increase with respect to the SFE value of 22 ± 4 mJ·m^−2^ reported for the undoped CoCrNi alloy [21].
- The higher value of the SFE due to N doping, compared to interstitial-free CoCrNi, can hinder nucleation and growth of deformation twins and, thus, rationalize the lower fraction of deformation twin boundaries in the plastically deformed CoCrNiN alloy.
